# Identifying and Exploring Jean Watson’s Theory of Human Caring in Nursing Approaches for Patients with Psychoactive Substance Dependence in Medical and Surgical Acute Wards

**DOI:** 10.3390/nursrep14030162

**Published:** 2024-08-28

**Authors:** Felice Curcio, Marzia Lommi, Rosa Nury Zambrano Bermeo, Ana Alejandra Esteban-Burgos, Gianluca Pucciarelli, Cesar Iván Avilés González

**Affiliations:** 1 Faculty of Medicine and Surgery, University of Sassari (UNISS), Viale San Pietro 43/B, 07100 Sassari, Italy; 2 Department of Biomedicine and Prevention, University Tor Vergata, 00133 Rome, Italy; marzia.lommi@uniroma1.it (M.L.);; 3 School of Health, Universidad Santiago de Cali, Cali 760000, Colombia; rosa.zambrano00@usc.edu.co; 4 Department of Nursing, Faculty of Health Sciences, University of Jaen, 23071 Jaen, Spain; aesteban@ujaen.es; 5 Instituto de Investigation Biosanitaria de Granada (Ibs.granada), University of Granada, 18012 Granada, Spain; 6 Department of Medical Sciences and Public Health, University of Cagliari, 09042 Cagliari, Italy; cesari.avilesg@unica.it; 7 Department of Nursing, Universidad Popular del Cesar, Valledupar 200002, Colombia

**Keywords:** human caring science, nursing, qualitative research, social discrimination, stigma, substance use disorder

## Abstract

Patients with substance use disorders may view healthcare professionals as capable of supporting them through their suffering and experience. Although numerous studies have focused on the roles, approaches, and attitudes of healthcare professionals, there is a lack of information on the nursing perspective. This study aims to explore the experiences and nursing approaches towards patients with psychoactive substance dependence admitted to an Italian acute hospital ward. A qualitative phenomenological study was conducted. Data were collected using semi-structured face-to-face interviews. The interviews were transcribed, read thoroughly, and analysed. Fifteen nurses were interviewed. Six main themes were extracted: (1) origin context, (2) participants’ personal thoughts, (3) type of approach provided, (4) school education received on the topic, (5) effectiveness of rehabilitative therapy, and (6) methods that can improve nursing care. The findings suggest that most respondents experience stigma and discrimination when providing care to these patients. In response to the results obtained, models have been suggested, such as Jean Watson’s Human Caring Theory, which shows how practising a holistic approach based on empathy and active listening can improve the relationship between nurses and patients. Furthermore, to eliminate stereotypes, it would be appropriate to act on the university education of nurses. This study was not registered.

## 1. Introduction

Findings from the 2024 World Drug Report highlight the continued expansion of drug markets [1]. The estimated number of people who have used a drug in the past 12 months continues to grow, reaching 292 million worldwide in 2022. Approximately 1 in 18 people aged 15–64 worldwide used drugs in the past 12 months, and one in 81 have a drug use disorder. One in four persons who have used any drug in the past year is a woman [1]. 

In Italy, analysis of 2022 Hospital Discharges (SDOs) showed 6555 hospitalisations for drug use issues, up slightly from the previous year, with 12 hospitalisations per 100,000 residents [2,3]. The social and health impact is evident not only because of the premature mortality rate but also because of poor health due to disabilities. According to the latest WHO estimates, in 2019 alone, psychoactive drug use resulted in an estimated 36.7 million DALYs and almost 600,000 deaths worldwide [4]. However, drug addiction treatment and health service provision remain inadequate, with only one in six people suffering from drug use disorders receive treatment [1].

In 2023 in Italy, 9336 people receiving assistance had at least one psychiatric pathology, of which 58% suffered from personality and behavioural disorders, 13% from neurotic and somatoform syndromes, 13% from schizophrenia and other functional psychosis, 3% from depression, 2% from mania and bipolar affective disorders [2]. Individuals with a dual diagnosis, substance use disorder, and other psychiatric disorders, have greater clinical and psychosocial severity and are more likely to engage in illicit behaviours than those with a substance use disorder. Consequently, the coexistence of mental disorders and substance use disorders has increasingly become a critical concern.

According to the Diagnostic and Statistical Manual of Mental Disorders, substance use disorder is classified as “addictions and related disorders” and is defined as “a pathological pattern of substance use that leads to clinically significant distress or impairment occurring within a 12-month period” [5]. Psychoactive substances are defined by the WHO as substances that, when consumed or administered, can change consciousness, mood, or thinking processes. Whereas sometimes boundaries of what is considered as “psychoactive” are difficult to delineate clearly, there is a common recognition of the main groups of psychoactive substances, including alcohol, nicotine, opioids, cannabis, cocaine, amphetamines, and other stimulants, hallucinogens, hypnotics, and sedatives [4]. Patients with psychoactive substance addiction problems often see healthcare professionals as a source of support, helping them cope with their suffering and intense emotional experiences. However, those needs may not be explicitly expressed as such but may often hide behind aggressive behaviours and/or defensive mechanisms. Despite the complexity of the situation that can make it difficult to approach a person, nurses must uphold their roles as caregivers, experts, communicators, and health promoters [5]. Within the healthcare profession, although the primary goal is to improve the health of patients, achieving this goal can be particularly challenging in the addiction context. In the study conducted by Ramos et al. [6], a critical aspect is the lack of training and experience on the new psychoactive substances that are emerging, making it even more difficult to provide health care in a clinical medical and addiction context. Another critical factor, as highlighted by Stigliano et al. [7], is the inappropriate use of opioids, which cause significant public health problems. This leads to reflection on a dynamic and continuous approach to the training of health personnel, which is made difficult by a lack of resources and administrative strategies where traditional or already known health interventions are not sufficient.

From a nursing perspective, where caring is the essence of professional practice, it is critical to take a holistic approach that addresses not only the biological but also the emotional and psychosocial needs of patients [8]. Jean Watson’s Theory of Human Care provides a vital framework for understanding and improving the quality of care in these complex situations. Watson emphasises the importance of a nurturing relationship between patient and healthcare professional, proposing a model of care that blends scientific knowledge with a deep ethical and humanistic responsibility [9,10,11].

Nurses must be able to manage their frustration and maintain a trusting relationship with the patient, regardless of the difficulties encountered. It is critical that patients do not adopt negative or evasive attitudes that hinder their response to their needs. The literature highlights how often negative perceptions of patients with substance use disorders can significantly influence their management [11]. Therefore, it is imperative that all practitioners, including those in acute wards who do not specialise in the management of individuals with substance abuse disorders, employ effective treatment approaches that incorporate biological, behavioural, and social context elements, involving both medical and psychosocial support [12,13].

This study aims to examine whether Jean Watson’s theory of humanised care is reflected in the experiences of nurses serving in acute hospital wards that do not specialise in addiction treatment, and to evaluate current nursing approaches.

## 2. Materials and Methods

### 2.1. Design

The concept for this study emerged from the training experiences of nursing students who noticed that many patients hospitalised in medical and surgical wards, in addition to dealing with physical health issues, also had dependencies on psychoactive substances. This study was conducted using interpretative phenomenological analysis, a qualitative research approach that values ‘a detailed experiential account of the person’s involvement in the context’ [14]. Interpretative phenomenological analysis (I.P.A.) enables an understanding of the meanings of communication through the narration of participants’ experiences from cultural, social, and personal perspectives [15,16]. Interpretive phenomenological analysis develops into a double hermeneutic circle. On one hand, it adopts an idiographic approach because of the individual case investigation; on the other hand, it uses an interpretive approach, following the principles of Husserlian hermeneutic phenomenology [17].

The study adheres to the Consolidated Criteria for Reporting Qualitative Research (COREQ) [18].

### 2.2. Sampling and Recruitment

All nurses serving in the acute medical and surgical units at the study hospital were invited by the research team to participate in the study. During the recruitment phase, the internal medicine ward had 21 nurses, 8 of whom participated in the study, while the medical-surgical ward had 17 nurses, with 7 of them participating. This detailed sampling strategy aimed to ensure that participants had relevant experiences with patients suffering from both physical health issues and substance use disorders. This intentional sampling, performed by the authors FC and CIAG, allowed us to explore the nursing experiences and approach toward patients with psychoactive substance dependence admitted to medical and surgical wards.

Enrolment of participants lasted until data saturation was reached (n = 15). None of the recruited persons refused to participate in the study. Thus, it can be said that the method implies an interpretative approach enriched by descriptive notes.

### 2.3. Data Collection

Participants were invited by e-mail, and it was ensured that only the researcher and respondents were present during the interview with nurses who had experience working with patients in these wards, particularly those with substance use dependencies, despite the primary reasons for hospitalisation being related to physical illnesses. This focus was reinforced by the first question of the semi-structured interview included in Appendix A: “Tell us about your experience with a patient who had a substance use disorder”. Data were collected using semi-structured, face-to-face, and individual in-depth interviews. This type of interview was chosen because it is particularly informative, allowing the researcher to create a framework for the topics covered. The semi-structured interview guide provides a clear set of instructions for interviewers and, at the same time, can provide reliable and comparable qualitative data [14].

After reading the relevant literature, the researchers developed a 13-question semi-structured questionnaire (Appendix A). Three nurses, experts in advanced practice, and qualitative research validated the content and wording of the interview guide. Probing and clarifying were asked during the interview to ensure that rich data information was obtained [19].

### 2.4. Data Analysis

The average duration of the interviews was 25 min. The interviews were audio-recorded, replayed several times, and transcribed in full by assigning identification numbers. Each interview was independently and thoroughly read and re-read by two interviewers, initially noting the descriptive, linguistic, and conceptual elements that emerged from the text [20]. Next, emerging themes were identified, organised in a table for comparison, and finally grouped within superordinate themes. Consensual validation was performed between the two researchers, with no disagreements emerging.

### 2.5. Rigour

The study’s rigour was achieved by applying the criteria for readability, transferability, dependability, and conformability recommended by Lincoln and Guba [21]. Data were used and transcribed without comments and as direct quotes from semi-structured interviews. The inclusion/exclusion criteria, participant characteristics, context, data collection, and analysis procedures were detailed [22]. To minimise the risk of confirmation bias, the quotes were shared among the researchers, and finally, the results were shared with the participants.

### 2.6. Ethical Considerations

This study was conducted in line with the ethical guidelines for research in the Declaration of Helsinki [23], the Italian privacy law (Decree No. 196/2003), and the General Data Protection Regulation (GDPR-EU 2016/679). The interviews were authorised by the hospital institutional review committee and all participating nurses. Each participant understood the purpose of the study, that participation was voluntary, that anonymity and confidentiality of data would be assured by the author CIAG, and finally, informed consent was acquired. The ability to withdraw from the study at any time was also guaranteed. Each interview was assigned an alphanumeric code with no possibility of identifying the participants.

## 3. Results

The data analysis identified six main themes: (1) origin context, (2) personal thoughts of participants, (3) type of approach provided, (4) school education received on the topic, (5) effectiveness of rehabilitative therapy, and (6) methods that can improve nursing care.

### 3.1. The Origin Context

The nurses interviewed came from acute wards, medicine, and surgery, therefore non-specialist settings for the management of patients with psychoactive substance addiction.

### 3.2. Personal Thoughts of Participants

To assess this important topic, a series of questions were asked about experience with patients suffering from addiction, emotional impact of caring for a patient with substance dependence, and aspects that most impressed the interviewee after caring for a patient with a substance use disorder. These questions brought about an important dualism among some interviewees. On the one hand, we have the nurse who, as a professional, declares to “have no prejudices” (I_1) in caring for these patients because they are “people like any other, so they need to be cared for” (I_14), on the other, we have the anthropic aspect, where it is stated that “as a human being I cannot understand the life choices of these patients” (I_14). Those who find themselves with this kind of prejudice state that these patients are people who “seek death” (I_2). Consequently, according to these respondents, “patients with substance use disorders should pay for their own health care, just as they spend money to buy these substances” (I_7).

### 3.3. The Type of Approach Provided

For more than one question, nurses’ approaches to treating patients with substance use disorders were investigated. The answers varied, but the majority of the interviewees emphasised the difficult management of this group of patients in a non-specialist ward, evidenced by the fact that their presence disrupts the whole ward’ (I_8); this was motivated by the fact that this type of patient lies, always lies’. Most of the interviewees agreed that these patients have a “great manipulative capacity” and that “if you are slightly accommodating, they immediately understand and try to guide you towards what they want” (I_9). According to some, “you should never give too much confidence to these patients” (I_1 and I_2), thus summarizing the patient as “the one who must be distrusted” (I_1).

The act of lying may sometimes be accompanied by “threats” (I_7) and “attacks, not only verbal but also physical” (I_2).

Compared with other patients, patients with substance use disorders are described as not abiding by the rules of the ward (“he behaved as he wanted, he did not respect the ward hours, he wanted to eat when he felt like it, take drugs when he felt like it, he tried to trample on our person,” I_10). Furthermore, some nurses reported that “attempts to help are useless because they do not listen” and “they do what they want” (I_3). Nurses who assisted patients with these attitudes reported that they “wasted a lot of their time” and that “everything was an excuse to slow down work” (I_1). From what has been reported thus far, it is clear that a barrier is created between health workers and patients that does not allow them to actively listen to and consequently understand the patient’s condition. Some interviewees admit to being “prejudiced” (I_6) and think that patients have “difficulty relating to nurses, for fear of being judged”, “health workers give up relating as much as patients do” (I_4). Among the reasons for these attitudes is emphasised by one nurse (I_10) that “the time you spend with these patients is time wasted” and this “is a bit discouraging; this discouragement, of not seeing any improvement, leads us to give up”.

Nurses who assumed that these patients “had no desire to go beyond their psychoactive substance addiction” found themselves “arguing heavily” with these patients and taking a different approach to them than they usually do with non-substance-abusing patients (I_11).

As one of the most important principles of physics reminds us, ‘for every action there is an equal and opposite reaction’, I_8 affirms it in the same way: if one feels ‘aversion towards these patients, obviously the patient feels it’ and behaves accordingly.

Similarly, some interviewees, albeit in the minority, recall the mandate of the nurse, who, although working in a ward that is not specialised in the management of patients with addiction disorders, “should not only be concerned with the diagnosis written in the medical record but should take time to care, sit down and have a dialogue with the patient, understand his or her state of mind and initiate a pathway of awareness that can benefit the patient” (I_10).

### 3.4. School Education Received on the Subject

**Figure 1:**
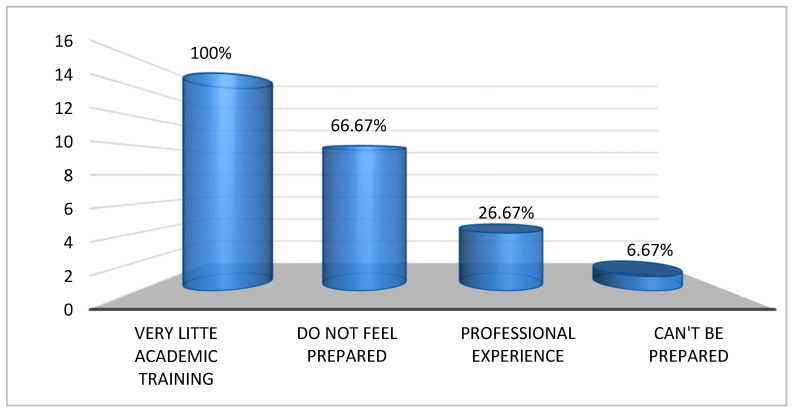
Academic training related to the provision of nursing care to patients with psychoactive substance dependency.

The interviewees were asked how they perceived their level of preparedness with respect to providing nursing care to patients with psychoactive substance dependence. The answers can be summarised schematically as follows (Figure 1):

- 10 out of 15 nurses admit that they “do not feel prepared”.
- 4 out of 15 nurses state that what they have learnt comes mainly from “professional experience” rather than from the course of study.
- 1 out of 15 nurses state that because these patients are unpredictable, “you cannot be prepared” (I_5).

In particular, all of the nurses interviewed emphasised that, although academic training on the topic did take place, it was very little, and the topic was approached more “from a pharmacological than a psychological point of view” (I_7).

### 3.5. The Effectiveness of the Rehabilitation Therapy

In the interviews with nurses, they were asked about their perception of the effectiveness of rehabilitation treatments offered to patients with addictions to psychoactive substances. Many of the interviewees agreed that “pharmacological treatment must be in synergy with behavioural treatment” (I_4), highlighting the need for a comprehensive approach that not only addresses physical aspects but also behavioural aspects of dependency.

In addition, a critical aspect was identified by the interviewees in relation to the failures of the support networks of the health system, both within and outside the hospital. According to a nurse, “the hospital network is ineffective, almost non-existent, as is a solid community network” (I_9), which makes it difficult to achieve long-term rehabilitation objectives. Another nurse added: “without effective community support, treatment loses its impact once the patient leaves the hospital environment” (I_7).

This perception was echoed by several interviewees that the lack of continuity in care and the limited resources in extra-hospital environments create significant obstacles to the rehabilitation and recovery of the patient. “Although we do what we can within the hospital, without a support structure in the community, our interventions are truncated” (I_12).

In summary, these results in relation to the effectiveness of rehabilitation therapy emphasise the importance of treatment not only at the time of hospitalisation but also in creating solid support networks in the territory, which strengthens hospital efforts and provides more possibilities for effective and sustained rehabilitation over time.

### 3.6. Methods That Can Improve Dedicated Nursing Care

Nurses were asked how they thought their work could improve the rehabilitation pathway of a patient with a substance use disorder in a non-specialised ward, such as medicine or surgery. The responses can be summarised as follows:

- 1/3 of the respondents stated that this aspect does not link directly and that a rehabilitation pathway should be created in the community and not in a non-specialised addiction ward (I_7).
- the remaining 2/3 of the interviewees reported that “a psychological pathway” or any kind of “support” is missing and that the only support these patients receive is “only pharmacological” (I_5).

Another four nurses suggested the presence of a specialised figure, such as a psychologist, to support the nurses at certain times when working with these patients, as they admit to feeling unprepared when dealing with this type of user.

## 4. Discussion

The present study aimed to explore the nursing experience and approach toward patients with psychoactive substance dependence admitted to acute hospital wards, and whether Jean Watson’s theory of humanised care is reflected in the experiences of nurses (Table 1 and Table 2) [24,25].

Analysis of the interviews shows that among nursing staff, there is still a form of stigma and discrimination in the care provided to patients with psychoactive substance addiction. This results in a greater need for the healthcare professional to be aware of his or her role; that is, to care for the person by taking into account his or her needs, listening to his or her needs and interests, and representing a reference person in the context of care [26,27,28]. As reported in the study “Barriers to access to general health and social services: a qualitative evaluation study on injecting drug users” [29], it follows from the biases of healthcare staff that the patient perceives these attitudes as “contempt for them, which leads the patient to feel less worthy of receiving care. As a result, a barrier is created due to the fact that nursing attitudes reflect society’s views, which label the addicted person even before he or she is given the opportunity to disprove the stigma. In this regard, Corley and Goren [30], argue that nurses expect a certain type of behaviour, such as that patients are “demanding, impatient, or that they can be “difficult”. Consequently, the patient notices this stigma and reacts by behaving exactly as the nurse expected [31]. This is also confirmed by other research, such as the studies by Francis et al. (2020) and Gunasekaran et al. (2022), which analysed the behaviour of mental health professionals dealing with dual-diagnosis patients. It has also been identified that negative expectations can influence the ability and attitude of patients, resulting in a cycle of behaviour that reinforces the initial preconceptions or prejudices of health professionals [27,28,32,33].

Most interviewees highlight the difficult management of this group of patients in a nonspecialist ward and claim that these patients have a “great capacity for manipulation” and that “one should never give too much confidence but should be wary.” This aspect is also reflected in the literature; in fact, a study conducted in Israel [34] points out that nurses are reluctant to provide care to patients who abuse drugs because they fear being manipulated by them. This finding is also echoed in a study exploring the patient's perspective. In the study in question, conducted in Ireland, three-quarters of patients reported being manipulated by healthcare staff, and nearly one-third reported lying and withholding information [35]. However, we should not be surprised if these patients tend to lie and manipulate staff; instead, we should strive to understand why they do so. Among possible motivations, studies by Dion (2024) and Reyre et al. (2014) point out that this type of behaviour could be due to previous experiences of stigmatisation and lack of trust from healthcare staff. Therefore, these lead to the development of manipulative behaviour or lies to protect oneself from new negative experiences in the healthcare environment [36,37].

The causes of this current stigma can be attributed to several factors, including insufficient training on the topic, workload that does not allow full dedication to patients, absence of professional support figures such as psychologists, lack of protocols and guidelines that can support healthcare workers, lack of awareness of the stigma itself, and past negative experiences. Beyond the personal traits that differentiate each person’s actions, it is important for health professionals, in this case nurses, to respect their role, which is to provide care without discrimination, as established by the code of ethics. These aspects are confirmed in the studies conducted by Daibes et al. (2017), Ramos et al. (2020), and finally Da costa et al. (2019), who emphasise the importance for nurses to put Jean Watson’s Caritas process into practice, stressing that care should be without prejudice and respectful of the individual’s dignity [6,38,39].

During the various interviews, some nurses reported positive approaches, such as deeming individuals with substance abuse disorders equal to other individuals, and therefore deserving of care. Nurses inclined toward this thinking highlighted how an approach based on the practice of active listening can benefit the nurse-patient relationship and improve the patient’s hospital stay. This can be confirmed in different studies, such as Perkins (2021), da Costa et al. (2019), and Vrbnjak et al. (2023), in which active listening and authentic nurse-patient presence contribute to a relationship free of prejudice and mutual respect, also promoting job satisfaction for nurses and increasing positive experiences for patients [24,39,40]. This type of approach is based primarily on the practice of active listening, one of the values that recur in Jean Watson’s Theory of Human Care. In Watson’s holistic theory, notions of empathy and the ability to experience play important roles. That is, the ability to understand another person’s perceptions and feelings and to communicate them in a sincere, honest, and authentic way.

In addition, the notion of non-possessive warmth recurs, i.e., an attitude of closeness demonstrated through a moderate tone of voice, appropriate facial expressions, and a consequent relaxed and open posture [41]. Watson criticises the technological turn in contemporary medicine, which sees that nurses respond more to the demands of machines than to the needs of patients. According to theorists, nursing aims to promote health, prevent illness, cure the sick, and restore well-being [9,40]. In addition, nursing should aim to help people achieve a high level of harmony with themselves, foster self-knowledge and self-healing, and deepen the meaning of life. Interviewees who have adopted this approach to some extent, even through the practice of active listening, say that all the critical issues reported earlier are alleviated, and that the overall situation tends to improve.

As highlighted in the literature, the role of the communicator is emphasised as a basis for creating a relationship of trust between health workers and patients, specifically in patients with dependencies on psychoactive substances. Effective and efficient communication, in addition to enhancing the therapeutic relationship, reduces the impact of harmful elements such as inconsistency, denial, and mistrust, which can impede the relationship and, therefore, result in the recovery of patients with psychoactive substance withdrawal disorders. According to Kimberly Dion [36], substance abuse patients continue to receive poor quality care, resulting in little or no emotional and psychosocial well-being; for this reason, it is here where the nurse as communicator and caregiver is essential, nursing beyond therapeutic interventions, has the skills to make significant changes in the lives of this type of patients with interventions in education and prevention, thus contributing to pacifying the deterioration caused by addiction to psychoactive substances [32,34,37,38,41].

In addition, the relationship created between the patient and the nursing staff not only helps improve treatment results, but also has a positive effect on nurses, stimulating a process of reflection that leads to job and personal satisfaction. According to the study conducted in England, “The therapeutic relationship: Dead, or merely impeded by technology?” [42] and the one conducted by Mitchell [41], when a genuine nurse-patient relationship is created, nurse become one of the foundations of therapeutic success. It is, therefore, essential that nurses receive training in communication skills and emotional support, promoting their role as influential motivators and agents of change and playing an essential role in the rehabilitation of their patients [10,36,43].

These results must be considered in light of some limitations. First, the study was conducted in only one Italian hospital; therefore, the results may not be generalisable to other contexts. Moreover, voluntary participation in this study produced a small sample. However, we assume that the information power of the data is rich [44]. Second, the study did not investigate the previous work experience of the nurses interviewed, let alone their personal and educational experiences in the management of this type of patient. Such information may have influenced the results of this study. Thirdly, the gender variable was not collected. This may have generated a sex bias in the interpretation of the results obtained. For future studies, it is recommended to collect this variable in order to understand whether gender biases are present and how they affect the nursing care of patients with psychoactive substance dependence.

In conclusion, in order to promote mental health through the application of Jean Watson’s Theory of Human Caring, our findings offer valuable insights for nurses caring for patients with psychoactive substance dependence in acute medical and surgical wards (Table 2).

## 5. Conclusions

In conclusion, our findings offer valuable insights for nurses caring for patients with psychoactive substance dependence in acute wards, encouraging them to explore their own attitudes, beliefs, and expectations without any bias.

This study highlights an important duality among interviewees. On the one hand, we have nurses who state that they have no bias and recognise that these individuals need to be cared for, while on the other hand, we have nurses who emphasise the difficult management of this group of patients, stressing that they do not understand the life choices of these patients. Therefore, in order to change stereotypes, it is important to act on basic nursing education. In this regard, several suggestions have been made in the literature [33], among the most effective of which are attending classes taught by patients who use or have used drugs in the past and countering socially prevalent stereotypes, holding seminars aimed at identifying difficulties, addressing them, and providing the support needed to work with these patients. Furthermore, in university programmes, more time and attention should be devoted to the meaning of “holistic nursing” and its techniques in the nursing approach to the patient. Finally, another suggestion would be to take courses, including online and free of charge, whose purpose is to provide tools for healthcare professionals to improve nursing practices by fostering the integration of compassion and holistic care principles, as Jean Watson advocates [45,46].

## Figures and Tables

**Table 1:** Integration of Jean Watson’s Theory of Human Caring in nursing assessment for patients with substance use disorders.

Components	Interview Guide Questions	Connection to Watson’s Theory of Human Caring
Core Focus: Jean Watson’s Theory of Human Caring		Focuses on health promotion, human-centred care, empathetic communication, and establishing trust.
Experience with patients	1, 9, 10	Evaluation of how nurses apply empathetic care principles in daily practice.
Personal and emotional perceptions	2, 4, 5	Identification of emotional barriers affecting comprehensive and empathetic care.
Preparation and education	7, 8	Importance of training in caring competencies according to Watson, to enhance care quality.
Effectiveness of therapy and care improvement	12, 13	Emphasises the need for integrated treatments (pharmacological and behavioural) and a supportive, trust-based environment.
**Identified main themes in results**		
Origin context (3.1)	Impact of non-specialised care settings on the quality of human caring.	
Personal thoughts of participants (3.2)	Dualism in nurses’ perceptions and how these perceptions affect the implementation of human-centred care.	
Type of approach provided (3.3)	Assessment of empathetic care approach versus control and distrust approaches.	
School education on the topic (3.4)	Deficiencies in academic training affecting the ability to provide holistic human care.	
Effectiveness of rehabilitative therapy (3.5)	Need for a comprehensive therapeutic approach combining pharmacological care with emotional support.	
Methods to improve nursing care (3.6)	Proposals to strengthen human-centred care, including continuous training and specialised support.	

Table Footnote: This table illustrates the integration of Jean Watson’s Theory of Human Caring in the assessment of nurses working with patients with substance use disorders. It maps key interview questions and themes identified with Watson’s theoretical framework, highlighting the importance of empathy, holistic care, and the need for continuous education and support in nursing practice [24].

**Table 2:** Promoting health through the application of Jean Watson’s Theory of Human Caring in nursing practice for patients with substance use disorders.

Health Promotion Aspect	Nursing Interventions	Connection to Watson’s Theory of Human Caring
Empathetic communication	Active listening and providing non-judgemental support to patients.	This reflects Watson’s emphasis on creating a bond of trust and understanding in the nurse-patient relationship.
Holistic care	Integration of behavioural therapy with pharmacological treatment.	Emphasises treating the patient as a whole, addressing both physical and emotional needs.
Patient empowerment	Involving patients in decision-making about their care.	Supports Watson’s idea of respecting patients’ autonomy and empowering them in their care journey.
Building trust	Consistent and reliable interactions, minimising distrust	Promotes a caring environment where trust is the foundation of the therapeutic relationship.
Supportive environment	They create a safe space for patients to express their feelings and concerns.	Watson’s theory encourages a healing environment that fosters emotional and psychological well-being.
Preventative education	We are educating patients on the risks of substance use and promoting healthier lifestyles.	Aligned with Watson’s focus on health promotion and disease prevention as crucial nursing roles.
Reflective practice for nurses	Encouraging nurses to engage in self-reflection to improve care delivery.	Watson’s theory includes caring for oneself as an essential component of being able to care for others effectively.
Continuous support beyond hospitalisation	They are collaborating with community resources to ensure ongoing care after discharge.	Reinforces Watson’s holistic approach, ensuring continuity of care in and out of the hospital setting.

Table Footnote: This table outlines how applying Jean Watson’s Theory of Human Caring in nursing practice for patients with substance use disorders promotes health. The table highlights the vital nursing interventions in the study. It links them to the core principles of Watson’s theory, emphasising the importance of empathetic, holistic, and patient-centred care in promoting positive health outcomes [25].

## Data Availability

The data presented in this study are available from the corresponding author upon request.

## Public Involvement Statement

No public involvement in any aspect of this research.

## Guidelines and Standards Statement

This manuscript was written following the recommendations of the COREQ guide for qualitative research reporting (Tong, 2007).

## Appendix A. Interview Guide

(1) Tell us about your experience with a patient who had a substance use disorder.
(2) What do you think of a patient who, in relation to the pathology for which he or she was admitted to the ward, has a second diagnosis of substance dependence?
(3) How do you think patients with a substance use disorder are managed on the ward?
(4) What is your greatest concern/criticism about these patients?
(5) Identify the emotional impact that occurs when caring for a patient with a substance use disorder.
(6) What attitudes do patients with a substance use disorder have towards you?
(7) What is your preparation to assist a patient with a substance use disorder? How do you qualify your preparation in terms of course of study?
(8) What is your educational background in the field of substance use disorders?
(9) What is the thing that most impressed you after caring for a patient with a substance use disorder?
(10) What memories do you have of caring for a patient with a substance use disorder?
(11) How did you feel when you entered alone the room of a patient with a substance use disorder?
(12) What do you think about the effectiveness of therapy for people with a substance use disorder?
(13) How do you think your work can improve the rehabilitation of a patient with a substance use disorder?
